# Lung Recruitment Can Improve Oxygenation in Patients Ventilated in Continuous Positive Airway Pressure/Pressure Support Mode

**DOI:** 10.3389/fmed.2015.00025

**Published:** 2015-04-21

**Authors:** András Lovas, Márton Ferenc Németh, Domonkos Trásy, Zsolt Molnár

**Affiliations:** ^1^Department of Anaesthesiology and Intensive Therapy, Faculty of Medicine, University of Szeged, Szeged, Hungary

**Keywords:** capnography, CPAP ventilation, lung compliance, lung recruitment, positive end-expiratory pressure

## Abstract

**Background:**

Recruitment maneuvers are often used in critical care patients with hypoxemic respiratory failure. Although continuous positive airway pressure/pressure support (CPAP/PS) ventilation is a frequently used approach, but whether lung recruitment also improves oxygenation in spontaneously breathing patients has not been investigated yet. The primary objective was to analyze the effect of recruitment maneuver on oxygenation in patients ventilated in CPAP/PS mode.

**Methods:**

Following baseline measurements PEEP was increased by 5 cmH_2_O. Recruitment maneuver was applied for 40 s with 40 cmH_2_O of PS. Measurements of the difference in PaO_2_/FiO_2_ and airway parameters measured by the ventilator were recorded immediately after recruitment then 15 and 30 min later. Thirty patients ventilated in CPAP/PS mode with a PEEP ≥5 cmH_2_O were enrolled in this prospective, observational study if their PaO_2_/FiO_2_ ratio was <300 mmHg or required an FiO_2_ >0.5.

**Results:**

Following recruitment maneuver patients were considered as non-responders (NR, *n* = 15) if difference of PaO_2_/FiO_2_ <20% and responders (R, *n* = 15) if difference of PaO_2_/FiO_2_ ≥20%. In the NR-group, PaO_2_/FiO_2_ decreased non-significantly from baseline: median [interquartile], PaO_2_/FiO_2_ = 176 [120–186] vs. after recruitment: 169 [121–182] mmHg, *P* = 0.307 while in the R-group there was significant improvement: 139 [117–164] vs. 230 [211–323] mmHg, *P* = 0.01. At the same time points, dead space to tidal volume ratio (Vds/Vte) significantly increased in the NR-group Vds/Vte = 32 [27–37] vs. 36 [25–42]%, *P* = 0.013 but no significant change was observed in the R-group: 26 [22–34] vs. 27 [24–33]%, *P* = 0.386.

**Conclusion:**

Recruitment maneuver improved PaO_2_/FiO_2_ ratio by ≥20% in 50% of patients ventilated in CPAP/PS mode.

## Introduction

Hypoxemic respiratory failure is one of the leading causes of the need for mechanical ventilation and can occur in several conditions, most often as a result of heart failure, pneumonia, and sepsis. Its most severe form is acute respiratory distress syndrome (ARDS), which still carries high risk of mortality (1). Applying increased level of PEEP (2), prone positioning (3), and lung recruitment (4) are all recommended measures to improve oxygenation and gas exchange in general.

One of the main reasons of hypoxemia, characterized by low partial arterial oxygen tension/fraction of inspired oxygen (PaO_2_/FiO_2_), is diffuse atelectasis of the alveoli (5). Therefore, resolving atelectatic lung areas could reduce intrapulmonary shunt and venous admixture and hence improve oxygenation (6). This can be achieved by applying increased intrathoracic pressures for a short period of time and keep the alveoli open by titrating the adequate level of PEEP. This procedure of opening up atelectatic alveoli and keep them open is called as the “open lung concept” (7). However, it is also well known that not every lung responds for recruitment maneuvers (8). Although most recruitment strategies were tested under controlled mechanical ventilation (9), there is also increasing evidence that maintaining spontaneous breathing during mechanical ventilation may provide advantageous ventilation/perfusion ratio and prevents alveolar shearing (6, 10). Applying continuous positive airway pressure (CPAP) with or without pressure support (PS) and allowing the patient to breathe spontaneously is an often used ventilation mode, both during non-invasive and invasive ventilation. Although theoretically alveolar recruitment may also have a place in these patients ventilated invasively this has only been investigated during non-invasive ventilation (11). Sophisticated methods of assessing lung recruitment, like computer tomography (CT) scanning, esophageal pressure measurements, etc., are rarely available in the everyday routine in most countries and these are especially difficult to perform in spontaneously breathing patients (12). Nevertheless, one of the benefits of effective recruitment is improved oxygenation after the maneuver. Therefore, the purpose of the current study was to investigate the effects of recruitment on the PaO_2_/FiO_2_ ratio in patients ventilated in CPAP/PS mode suffering from moderate and severe hypoxemic respiratory failure.

## Materials and Methods

### Patients

Following ethics committee approval of the Human Investigation Review Board of University of Szeged, informed consent was obtained from the patients’ next of kin. All mechanically ventilated patients with maintained spontaneous breathing, ventilated in CPAP/PS mode with a PEEP ≥5 cmH_2_O, were enrolled in this prospective, observational study if their PaO_2_/FiO_2_ ratio <300 mmHg or required an FiO_2_ >0.5, regardless of the etiology of respiratory failure (13, 14). Exclusion criteria were age <18, pregnancy, pulmonectomy/lobectomy, or spontaneous pneumothorax in past-medical history, emphysematous bullae, clinically diagnosed end stage chronic obstructive pulmonary disease, and vasopressor refractory hemodynamic instability.

### Measurements and experimental protocol

All patients who were eligible for the study had a radial arterial and an internal jugular or subclavian central venous catheter inserted on admission to the intensive care unit as part of our standard care. Patients were sedated with continuous infusion of propofol and fentanyl reaching a Richmond Agitation Sedation Scale score of −1 to −2. Electrocardiogram, invasive blood pressure, and SpO_2_ were continuously monitored by Dräger Infinity Gamma XL Monitor (Telford, PA, USA). Patients were ventilated with Dräger Evita^®^ XL respirators (Lübeck, Germany). The level of PS was adjusted to achieve adequate arterial pCO_2_ level to maintain pH ≥7.30. Respiratory parameters, airway pressures, dynamic respiratory compliance, airway resistance, end-tidal carbon dioxide (EtCO_2_), dead space (Vds), and dead space to exhaled tidal volume ratio (Vds/Vte) were all continuously monitored by the respirator and its own volumetric capnography.

Once inclusion criteria were fulfilled respirator settings, cardio-respiratory and airway parameters were recorded at baseline. Then PEEP was increased by 5 cmH_2_O and after 5 min measurements were repeated to investigate the effect of any PEEP-induced recruitment. For alveolar recruitment, PS was increased to 40 cmH_2_O for 40 s to limit the undesirable side effects of volutrauma. After which peak inspiratory pressure was reduced to the initial value as at baseline while maintaining the increased level of PEEP (by 5 cmH_2_O) according to the open lung concept (7). Measurements were repeated immediately after recruitment then 15 and 30 min later with constant respirator settings as at baseline. Arterial blood gas samples were analyzed by a Roche cobas b 221 (Mannheim, Germany) blood gas system at each measurement points and central venous samples were taken at baseline and at the final time point to determine central venous oxygen saturation (ScvO_2_).

Primary outcome parameter was the change in oxygenation (PaO_2_/FiO_2_) after the recruitment maneuver. Patients were considered as non-responders (NR) if difference of PaO_2_/FiO_2_ <20% and responders (R) if difference of PaO_2_/FiO_2_ ≥20% between baseline and following recruitment measurements.

### Statistics

Based on a preliminary analysis of our data (15), the mean PaO_2_/FiO_2_ ratio before recruitment was 156 mmHg with a SD of 43 mmHg. In order the study to have an 80% power with a *P* < 0.05 and to observe an increase in the PaO_2_/FiO_2_ of 10 or 20% (which corresponds to a PaO_2_/FiO_2_ of 171 and 186 mmHg, respectively) after recruitment, the required minimal sample size was calculated to be 51 or 13. Therefore, we decided that a sample size of 30 should be feasible and provide adequate statistical power.

All data in the tables are presented as median [interquartile range]. Figures are presented as boxplots: 5th–95th percentile, interquartile range, and median. After testing for normality with Shapiro–Wilk test data were analyzed between groups with Mann–Whitney *U* test or Kruskal–Wallis test as suitable. Matched pairs were investigated with Wilcoxon signed rank test and relationship was analyzed with Spearman’s correlation coefficient. For evaluating goodness of fit and independence, Pearson’s chi-square test was used. The “*P*” value was considered significant if <0.05. For statistical analysis, IBM SPSS Statistics Version 20 (Armonk, NY, USA) software was used.

## Results

Over the study period 30 patients were enrolled, of whom 15 (50%) patients turned out to be NR and 15 (50%) responders. There was no significant difference between groups in baseline respirator settings and demographic characteristics except of age. Out of the 19 patients with admission diagnosis of cardiac origin 13 (68%) were responders (Table 1). Serious adverse effects of recruitment maneuver like pneumothorax and worsening hemodynamic instability were not detected.

**Table 1:** **Demographic data**.

	Non-responders (*n* = 15)	Responders (*n* = 15)	*P*
Age (years)	63 [55–58]	74 [59–76]	0.045
Male/female (*N*)	11/4	9/6	
Body-mass index (kg/m^2^)	27 [24–31]	29 [25–34]	0.389
APACHE II score	21 [18–25]	23 [19–33]	0.851
Baseline PEEP (cmH_2_O)	10 [8–12]	10 [10–12]	0.389
Baseline FiO_2_ (%)	60 [50–62]	60 [60–80]	0.126
Baseline PS (cmH_2_O)	12 [8–16]	10 [10–16]	0.935
Ventilated days (*N*)	4 [2–6]	2 [1–4]	0.202
Lung injury score	2.3 [1.7–2.7]	2.3 [2.0–2.8]	0.461
Orotracheal tube ID (mm)	8 [8–8.5]	8 [8–8]	0.567
Cause of admission (%)			
Heart failure	4 (13)	6 (20)	
Ischemic heart disease	2 (8)	7 (24)	
Pneumonia	3 (10)	1 (3)	
Sepsis	3 (10)	0	
Pulmonary contusion	1 (3)	0	
Stroke	1 (3)	0	
Other	1 (3)	1 (3)	

*APACHE II, acute physiology and chronic health evaluation II; PEEP, positive end-expiratory pressure; PS, pressure support; ID, internal diameter*.

**Figure 1:**
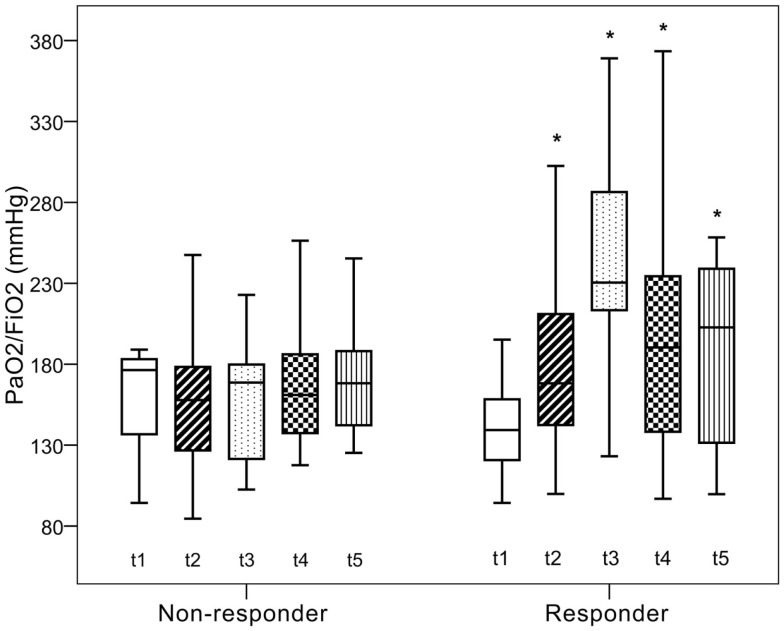
**Changes in PaO_2_/FiO_2_**. *t*_0_, baseline measurements; *t*_1_, increasing PEEP by 5 cmH_2_O; *t*_2_, after recruitment; *t*_3_ and *t*_4_, 15 and 30 min after recruitment. *Significant difference as compared to baseline measurements, *P* < 0.05.

There was a non-significant decrease in PaO_2_/FiO_2_ from baseline to 30 min following recruitment in the NR-group. In the R-group, PaO_2_/FiO_2_ significantly improved after the recruitment maneuver as compared to baseline results and remained elevated throughout the observation period. There was significant improvement in SaO_2_ among responders, while there was no significant change in the NR-group. Bicarbonate and base excess levels showed significant difference between groups at all time points. Hemodynamic parameters and ScvO_2_ did not show any significant change over time (Table 2; Figure 1).

**Table 2:** **Hemodynamic variables and blood gas results**.

	Group	Time point				
		Baseline	PEEP increment	After RM	15 min following RM	30 min following RM
Heart rate (1/min)	NR	88 [64–99]	89 [68–102]	87 [66–100]	91 [67–99]	90 [67–99]
	R	95 [70–100]	95 [72–115]	93 [70–106]	92 [70–101]	99 [70–119]
MAP (mmHg)	NR	75 [68–92]	80 [70–83]	79 [68–88]	79 [69–83]	80 [70–86]
	R	75 [69–88]	75 [71–87]	78 [66–87]	76 [68–85]	74 [69–86]
SaO_2_ (%)	NR	96 [93–99]	97 [94–99]	97 [94–99]	97 [95–98]	97 [95–99]
	R	95 [94–97]	96 [95–98]^a^	98 [96–99]^a^	98 [96–99]^a^	97 [96–99]^a^
PaCO_2_ (mmHg)	NR	47 [44–50]	50 [44–52]	48 [43–50]	48 [45–53]	48 [43–52]
	R	39 [37–49]	41 [37–50]	42 [37–51]	40 [37–52]	39 [37–53]
ScvO_2_ (%)	NR	74 [70–82]				77 [69–83]
	R	76 [69–79]				77 [72–81]
pH	NR	7.4 [7.3–7.4]	7.4 [7.3–7.4]	7.4 [7.3–7.4]	7.4 [7.3–7.4]	7.4 [7.3–7.4]
	R	7.4 [7.3–7.4]	7.4 [7.3–7.4]	7.4 [7.3–7.4]	7.4 [7.3–7.4]	7.4 [7.3–7.4]
${\mathrm{HCO}}_{3}^{-}$ (mmol/L)	NR	28 [24–31]	29 [24–31]	28 [24–31]	29 [24–31]	28 [23–31]
	R	23 [20–28]^b^	23 [21–27]^b^	23 [21–28]^b^	23 [21–28]^b^	23 [20–28]^b^
BE	NR	2.7 [−0.3 to 4.5]	3.2 [−0.7 to 5.1]	2.9 [−0.6 to 5.0]	3.2 [−1.3 to 5.3]	3.0 [−2.4 to 5.0]
	R	−1.4 [−4.2 to 2.5]^b^	−1.6 [−4.2 to 2.5]^b^	−1.8 [−4.2 to 2.1]^b^	−1.9 [−4.2 to 1.9]^b^	−1.9 [−4.2 to 1.8]^b^
Lactate (mmol/L)	NR	0.9 [0.7–1.1]	0.9 [0.7–1.0]	0.9 [0.7–1.1]	0.9 [0.6–1.0]	0.9 [0.6–1.0]
	R	1.1 [0.8–1.6]	1.1 [0.8–1.5]	1.1 [0.8–1.5]	1.1 [0.9–1.5]	1.0 [0.7–1.5]

*PEEP, positive end-expiratory pressure; RM, recruitment maneuver; NR, non-responder; R, responder; MAP, mean arterial pressure; ScvO_2_, central venous oxygen saturation; BE, base excess*.

*^a^Significant difference as compared to baseline measurements, *P* < 0.05*.

*^b^Significant difference between groups, *P* < 0.05*.

There was no significant change in tidal volume, Vte indexed for predicted bodyweight, respiratory rate, and minute ventilation between groups and throughout the study as compared to baseline parameters. In the NR-group dynamic compliance, a parameter indicated on the ventilator dropped non-significantly after the recruitment maneuver but there was a significant increase in Vds/Vte following recruitment and 15 min later as compared to baseline in the same group. There was no other significant change in the examined respiratory and airway parameters in the NR- and R-group (Table 3).

**Table 3:** **Respiratory and airway parameters complemented with arterial to end-tidal CO_2_ results**.

	Group	Time point				
		Baseline	PEEP increment	After RM	15 min following RM	30 min following RM
Vte (mL)	NR	473 [398–612]	479 [397–588]	447 [393–615]	506 [378–597]	471 [453–663]
	R	513 [406–667]	489 [385–702]	492 [398–602]	510 [354–698]	520 [402–741]
Vte/PBW (mL/kg)	NR	8 [6–8]	7 [6–9]	7 [5–8]	7 [6–9]	7 [6–9]
	R	7 [7–9]	7 [6–10]	7 [6–10]	8 [6–10]	8 [7–10]
RR (1/min)	NR	17 [13–22]	18 [13–20]	18 [13–20]	18 [13–20]	17 [14–22]
	R	19 [13–24]	19 [14–26]	17 [14–26]	19 [15–24]	18 [14–24]
MV (mL)	NR	7896 [7011–11,016]	8040 [6300–11,020]	7524 [7152–9825]	7809 [6230–10,380]	8208 [7260–10,296]
	R	9744 [8037–11,687]	9741 [8220–10,875]	9798 [7700–11,808]	10,101 [8328–11,328]	10,116 [8788–11,625]
*R*_aw_ (cmH_2_O/L/s)	NR	13 [9–14]	13 [9–18]	14 [10–17]	13 [9–18]	14 [9–18]
	R	11 [9–16]	11 [9–16]	11 [9–16]	11 [9–15]	11 [9–15]
*C*_rs_ (mL/cmH_2_O)	NR	68 [47–83]	65 [41–85]	64 [42–75]	69 [43–95]	68 [46–85]
	R	52 [34–98]	53 [31–106]	56 [36–90]	58 [39–98]	58 [39–99]
EtCO_2_ (mmHg)	NR	42 [34–45]	41 [35–47]	40 [34–45]	41 [36–47]	41 [37–45]
	R	36 [30–47]	37 [31–47]	39 [31–47]	38 [30–48]	39 [30–48]
P_(a-ET)_CO_2_ (mmHg)	NR	7 [4–13]	8 [4–10]	8 [5–13]	7 [4–11]	7 [4–13]
	R	5 [1–9]	6 [2–9]	6 [3–11]	5 [1–10]	6 [1–9]
Vds (mL)	NR	146 [128–191]	148 [135–201]	153 [133–198]	150 [127–198]	150 [127–198]
	R	153 [118–172]	166 [126–180]	144 [118–180]	153 [125–177]	158 [125–183]
Vds/Vte (%)	NR	32 [27–37]	35 [30–42]	36 [25–42]^a^	35 [29–41]^a^	32 [29–40]
	R	26 [22–34]	28 [24–38]	27 [24–33]	27 [24–34]	28 [25–36]

*PEEP, positive end-expiratory pressure; RM, recruitment maneuver; NR, non-responder; R, responder; Vte, exhaled tidal volume; Vte/PBW, exhaled tidal volume indexed to predicted body weight; RR, respiratory rate; MV, minute ventilation; *R*_aw_, airway resistance; *C*_rs_, dynamic compliance; EtCO_2_, end-tidal CO_2_; P_(a-ET)_CO_2_, arterial minus end-tidal CO_2_; Vds, dead space; Vds/Vte, dead space to exhaled tidal volume ratio*.

*^a^Significant difference as compared to baseline measurements, *P* < 0.05*.

**Figure 2:**
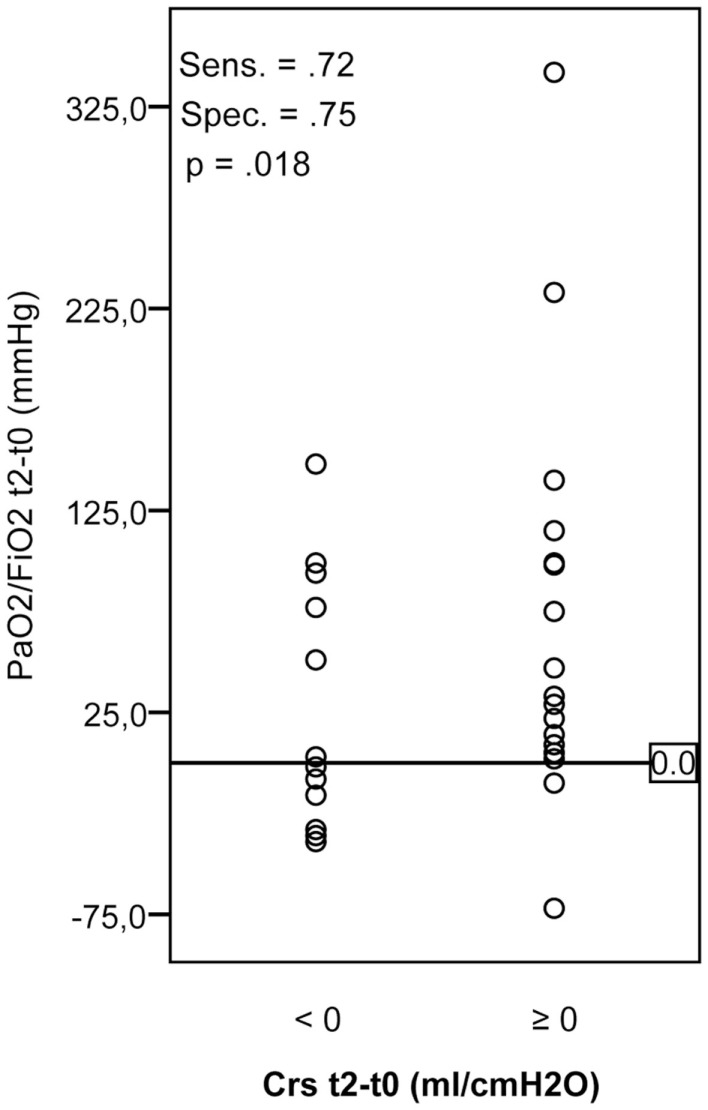
**Changes in PaO_2_/FiO_2_ and dynamic compliance after recruitment maneuver as compared to baseline parameters**. *C*_rs_, dynamic compliance, Sens., sensitivity; Spec., specificity.

Improvement in oxygenation was detected in 74% of all patients, and in 26% arterial oxygenation did not improve or even deteriorated. Testing in a contingency table the change in PaO_2_/FiO_2_ and dynamic compliance after the recruitment maneuver is shown in Figure 2. Improvement (≥0) or deterioration (<0) of dynamic compliance gave high sensitivity and specificity with a positive predictive value of 0.89 to differentiate patients with worsening as compared to those with improved PaO_2_/FiO_2_.

## Discussion

The most important finding of this study is that recruitment maneuver improved oxygenation by more than 20% in half of the patients with moderate and severe hypoxemic respiratory failure ventilated in CPAP/PS mode. We also found that patients in whom hypoxemia was due to cardiac origin seemed to benefit the most, as nearly 70% of these patients were found to be responders.

Patients, according to the change in the PaO_2_/FiO_2_ ratio after recruitment, were divided to non-responder and responder groups. Regarding the demographic data, it is an interesting finding that patients in the NR-group were significantly younger than those in the R-group. There were also more patients with ischemic heart disease and heart failure in the R-group, while there were only four patients with heart failure in the NR-group. One of the possible explanations is that although lung compliance decreases with age in general but success of recruitment depends on other factors like co-morbidities and it may be more successful in patients with heart disease as compared to patients with pneumonia. The beneficial effects of PEEP-induced alveolar recruitment with improved compliance and oxygenation are well known phenomenon in patients with ischemic heart disease (16). PEEP can also decrease intrapulmonary shunt such as hypoxic pulmonary vasoconstriction with a reduced pulmonary artery pressure among patients with heart failure (17). Therefore, it is not the age *per se* but the accompanying higher number of patients with heart condition that caused the observed difference in the current study. Our results draw the attention of the importance of the etiology of acute lung injury and co-morbidities, at least as far as improvement in oxygenation is concerned after the recruitment maneuver. These results are also in accord with those reported in patients on controlled mechanical ventilation (18) but it also contradicts those in which etiology did not seem to matter (19). However, in this recent study by Grasso et al., the sample size was small (11/group) and none of the patients were admitted due to acute heart failure. Nevertheless, the success of recruitment as far as oxygenation is concerned in spontaneously breathing patients having developed hypoxemic respiratory failure due to acute heart failure is an important finding and should be investigated further.

Although it is not the most accurate way to assess lung recruitment, but measuring changes in arterial oxygenation is one of the commonly used methods to detect the efficacy of recruitment (20, 21). Furthermore, there is no consensus on how to define responders and NR based on the PaO_2_/FiO_2_ values, which vary between 30 and 50% in the literature (19, 22). Due to the lack of well-defined values, we have chosen an arbitrary threshold of difference in PaO_2_/FiO_2_ ≥20% to define as responders and <20% as NR following recruitment. Nevertheless, we detected an improvement of oxygenation in 74% of all patients, and in 26% arterial oxygenation did not improve or even deteriorated. Taking the 20% improvement in oxygenation as a clinically significant change, 50% of patients still responded, which is similar to that of reported in recently published studies (23, 24). However, it is important to note that the ratio of responders is highly dependent on the defined threshold. Furthermore, the improvement in arterial oxygenation among responders lasted longer than in studies where controlled ventilation was applied. In the investigation by Oczenski et al., after the initial improvement, PaO_2_/FiO_2_ returned to the baseline values after 30 min (25) while in our trial the significant improvement in oxygenation persisted throughout, suggesting that the effects of recruitment may last longer in spontaneous assisted modes as compared to controlled modes of ventilation in hypoxemic respiratory failure. Although the sample size is too small for an outcome study, which holds true for all of the above mentioned investigations, but our data suggest that CPAP/PS ventilation and lung recruitment may have benefits in patients suffering from moderate to severe acute lung injury, especially due to acute heart failure, which should be investigated further.

It may also be important to note, that prior to intervention patients were ventilated for a median of 4 days in the NR-group while it was only 2 days in the R-group. Although it was not statistically significant, but these results are similar to that of reported by Grasso et al., where the length of mechanical ventilation was significantly shorter in those patients who responded for recruitment maneuvers (19).

It is well known that not every lung responds for recruitment and unnecessary maneuvers may lead to adverse effects (8, 26). Several methods had been evaluated of which chest CT scan remains the gold standard warranting the direct visualization of the recruitable lung tissue (8). However, this method requires the transport of the critically ill patients to the CT scanner and exposes them to radiation (27). Other bed-side measurements to assess recruitability are pressure–volume curve assessment and end-expiratory lung volume/functional residual capacity ratio measurement (28, 29). Unfortunately, due to financial and ethical reasons, these methods were not applied in our study therefore we only have limited proof on the change in lung volume after the recruitment.

We did not observe any significant change neither in the PaCO_2_ nor in any other blood gas variables throughout the study. However, there was a significant difference in bicarbonate and base excess levels between groups this observation had no effect on the investigation of recruitment. One of the potential alternatives for assessing alveolar recruitment may be the change in the difference between the arterial and end-tidal CO_2_ (P_a-ET_CO_2_) (30). In our study, P_a-ET_CO_2_ although did not change significantly over time in neither of the groups but in the R-group its value was lower than in the NR-group. Therefore, it may be a promising parameter but its relevance requires further studies.

Another important parameter is compliance, which is determined by volume/pressure relationships. Theoretically, in recruitable patients increasing pressures will increase volume hence compliance should improve or remain unchanged. While in non-recruitable patients increased pressures during recruitment can lead to the overdistension without gaining lung volumes, hence result in a consecutive fall in respiratory compliance (31). Although, in a recent study by Oczenski et al., in patients with ARDS who were ventilated in controlled mode and underwent recruitment after a PEEP trial there was no significant change in compliance 3 min after the maneuver what was accompanied by a significant improvement in oxygenation (25). This approach cannot be evaluated in our study as the value of the ventilator indicated compliance in spontaneously breathing patients has not been validated yet.

Finally, hemodynamic changes during the recruitment maneuver have been widely investigated (32, 33). Although we did not apply advanced hemodynamic monitoring in this study but as far as heart rate, mean arterial pressure, lactate, and ScvO_2_ are concerned there was no significant change after the recruitment procedure as compared to baseline therefore it is likely that patients remained hemodynamically stable, suggesting that performing recruitment maneuver in CPAP/PS ventilation is a safe strategy in patients with severe acute respiratory failure.

### Limitations

There are several limitations of our study. In the absence of lung CT scans, recruitment and the degree of the recruited lung area cannot be estimated. Although the investigation of Gattinoni et al. still remains the reference method to assess lung recruitment (8) we considered it difficult to be accepted ethically because of the potential dangers of transport and radiation. Furthermore, esophageal and herewith transpulmonary pressures were not monitored therefore we could not conclude if pleural pressure was swinging in spontaneously breathing patients hereby producing different recruitment effects during the time course of the maneuver. Finally, neither the sample size, which was too small, nor the protocol (with one single recruitment maneuver only) allowed us to draw any conclusion regarding hard clinical end-points such as ventilator free days, length of stay or outcome. However, based on the current findings, a study designed to answer these questions is certainly warranted. Nevertheless, the significant tendency what we observed has never been reported before and these preliminary results may provide important information for those who are interested in applying spontaneous assisted/supported modes of ventilation for patients with severe acute respiratory failure.

## Conclusion

Alveolar recruitment maneuver can improve oxygenation in patients suffering from moderate and severe acute hypoxemic respiratory failure and ventilated in CPAP/PS mode as indicated by the significant improvement in oxygenation after recruitment in 74% of all patients. The decrease in dynamic compliance as displayed on the ventilator after the recruitment maneuver proved to be a simple bed-side indicator of failure in improving oxygenation in spontaneously breathing patients.

### Author note

The results of this investigation were presented as a poster at the 35th International Symposium on Intensive Care and Emergency Medicine.

## Author Contributions

ZM and AL designed the trial, interpreted the results, and drafted the manuscript. MN gave substantial contributions to the conception and design of the study. AL carried out the statistical analysis. AL, MN, and DT participated in coordination and have made substantial contributions to analysis of data. AL, MN, and DT contributed with data collection and ZM, AL, MN and DT assisted in the critical revision of the manuscript. All authors have read and approved the final version of the manuscript.

## Conflict of Interest Statement

The authors declare that the research was conducted in the absence of any commercial or financial relationships that could be construed as a potential conflict of interest.
